# Blink reflex in newly diagnosed and treated patients with Wilson’s disease

**DOI:** 10.1007/s00702-021-02432-x

**Published:** 2021-10-20

**Authors:** Jan P. Bembenek, Karolina Kiryluk, Ewa Inglot, Tomasz Litwin, Łukasz Smoliński, Anna Członkowska

**Affiliations:** 1 Department of Clinical Neurophysiology, Institute of Psychiatry and Neurology, Sobieskiego 9, 02-957 Warsaw, Poland; 2 2nd Department of Neurology, Institute of Psychiatry and Neurology, Warsaw, Poland

**Keywords:** Blink reflex, Wilson’s disease, Magnetic resonance imaging, Basal ganglia, Cerebellum, Brainstem

## Abstract

Abnormal blink reflex (BR) results mainly from the dysfunction of reticular brainstem pathways and is one of the features of degenerative brain disorders. We aimed to investigate whether patients with Wilson’s disease (WD) have abnormal BR. This was a prospective, observational, single-center study. BR was assessed in accordance with generally accepted standards in 44 newly diagnosed treatment-naïve and 66 treated patients with WD. Any abnormal parameters in BR were observed in 45.5% treatment-naïve patients and 37.9% treated patients (*p* = 0.429). We also did not observe significant differences in BR parameters and frequency of abnormal findings between treated and treatment naïve patients. Abnormal findings in any of the BR parameters were more frequent in patients with neurological vs. non-neurological presentation (57.5 vs. 28.6%, *p* = 0.002), present vs. absent Kayser–Fleischer ring (73 vs. 21.5%, *p* < 0.001), and typical vs. no typical WD abnormalities in brain MRI (50% vs. 24.4%, *p* = 0.009). In addition, longer median R1 and R2 latencies, both ipsilateral and contralateral, were significantly more frequent in neurological than non-neurological WD patients, those with Kayser–Fleischer rings, and those with abnormal MRI findings typical of WD. Our results confirm frequent BR abnormalities in WD, which may be explained by the pathological influence of copper deposits in the circuit linking the basal ganglia, cerebellum and brainstem.

## Introduction

Wilson's disease (WD) is an autosomal recessive genetic disorder associated with abnormal copper metabolism, which is caused by mutations in the *ATP7B* gene, encoding a transmembrane copper-transporting ATPase. This results in pathological copper deposition in many organs and tissues, but particularly in the liver, brain and cornea (Kayser–Fleischer [K–F] rings), with subsequent secondary damage (Czlonkowska et al. 2018).

**Fig. 1:**
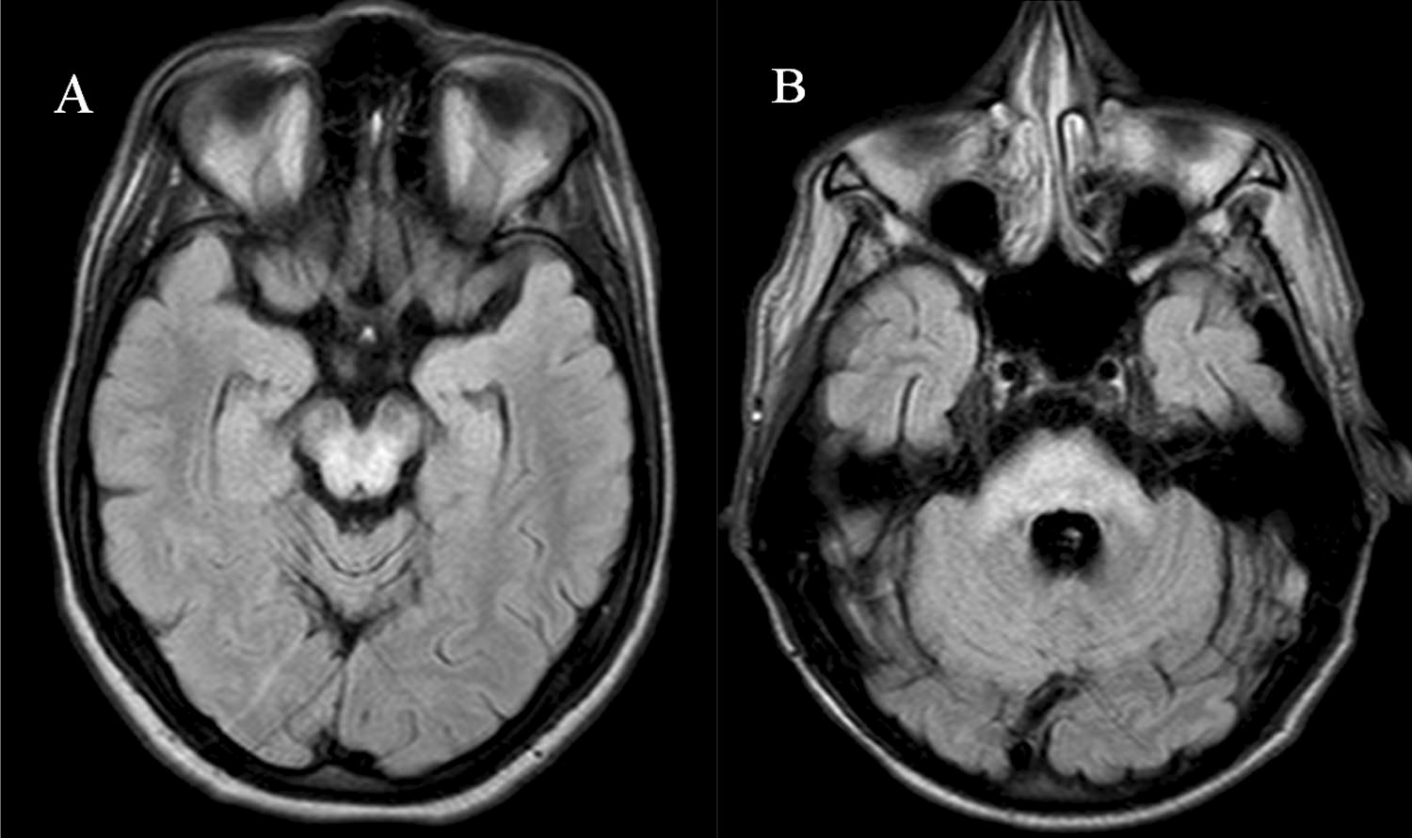
FLAIR image of hyperintensity in: **A** mesencephalon, **B** pons

Due to copper toxicity, many patients with WD have specific signs of pathology in the brain as detected by magnetic resonance imaging (MRI) (Dusek et al. 2019). The role of depositions of other ions like iron and manganese in the relevant brain structures is less explored (Litwin et al. 2013). Regions most often affected in WD are the basal ganglia, including putamen (72%), caudate nuclei (61%), as well as thalamus (58%), mesencephalon (49%), pons (20%), and cerebellum (10%) (Sinha et al. 2006). Abnormal hyperintense findings in fluid-attenuated inversion recovery (FLAIR) sequences of mesencephalon and pons in brain MRI are shown in Fig. 1.

Some studies suggest that the severity of brain atrophy may correlate with functional and neurological impairment in WD patients (Smolinski et al. 2019). Neurodegenerative changes are also very common—especially atrophy of the cerebrum (70%), brainstem (66%) and cerebellum (52%) (Czlonkowska et al. 2018; Sinha et al. 2006). Blink reflex (BR) is the best neurophysiological test to assess the excitability of the brainstem (Valls-Sole 2012).

The BR is a trigemino-facial polysynaptic reflex evoked by stimulation (e.g., electrical, mechanical, sound) of the supraorbital nerve, which results in bilateral contraction of the orbicularis oculi muscles. Most frequently, the BR is measured by applying an electrical stimulus in the orbital region and the reflex response of the supraorbital nerve is recorded by surface electrodes placed above the orbicularis oculi muscles. Two responses are recorded: R1 is the early ipsilateral response to the side stimulated and reflects function of an oligosynaptic pathway, while R2 is the late bilateral and polysynaptic response (Rushworth 1962; Ferrante 2018). As such, R2 may be more prone to interneuronal control by both segmentary and heterosegmentary influences (Holstege 1990; Ferrante 2018).

Trigeminal nerve sensory fibers conduct the afferent arc of the BR. In the efferent arc of the BR, impulses are conveyed through motor fibers of the facial nerves. An early R1 response terminates in mesencephalon. Late bilateral R2 responses are mediated by the spinal nucleus and tracts of trigeminal nerve. They reflect the connection of trigeminal and facial nerves and their nuclei in the lower medullary region by polysynaptic bilateral medullary pathways. R2 responses terminate in the facial nuclei (Cruccu et al. 2005). The most important external control of BR consists of an olivo-cerebellar circuit and dopaminergic system. Brainstem BR circuits can be modulated by basal ganglia via descending cortical projections or, alternatively, with input to the superior colliculus, via tecto reticular projections (Basso and Evinger 1996; Basso et al. 1996).

Based on the results of studies conducted with patients with Parkinson’s disease (Yavuz et al. 2015; Szmidt-Salkowska et al. 2016), multisystem atrophy (MSA), progressive supranuclear palsy (PSP) (Szmidt-Salkowska et al. 2016) and Huntington’s disease (de Tommaso et al. 2001; Valls-Solé et al. 2004), it may be assumed that abnormal BR will be observed in some patients with WD. Abnormal BR results can be caused by abnormalities in the cerebral cortex and in the basal ganglia (Esteban 1999), both of which are seen in WD. The current study aimed to investigate BR in patients with WD and relate any abnormalities to WD symptoms and MRI findings.

## Methods

### Patients

Between January 2017 and December 2020, patients aged ≥ 18 years with confirmed WD in accordance with the European Association for the Study of the Liver (EASL) Clinical Practice Guidelines (EASL 2012) were prospectively recruited at the 2nd Department of Neurology, Institute of Psychiatry and Neurology, Warsaw, Poland. Diagnostic methods of WD and clinical evaluation used in our center are described elsewhere (Członkowska et al. 2018). Other inclusion criteria included brain MRI provided up to 3 months prior to study recruitment.

Exclusion criteria included: significant involuntary movements in the face, enabling the examination of BR; the presence of abnormal findings in the brain MRI other than typical for WD; previous neurosurgical operations within the head; continuous use of neuroleptics, benzodiazepines, or antidepressants; disturbances in consciousness or the inability to understand the assumptions of the study and to give informed consent; pregnancy; medical history of the involvement of trigeminal and facial nerves or their nuclei that could affect the examined BR parameters; neuropathy; and acute liver insufficiency at study recruitment.

Patients with hepatic, neurological as well as the asymptomatic form of WD (detected during familial screening) were recruited. We compared BR parameters between treated patients and treatment-naïve patients who had not yet received any anti-copper treatment for WD. We also investigated any correlations between the presence of abnormal BR parameters and the form of the disease, abnormal brain MRI findings and the presence of a K–F ring.

The local bioethics committee approved this study and all patients gave informed consent.

### Brain imaging

All patients had routine brain MRI performed with either 1.5 or 3 T MRI scanner within one week of the BR evaluation. Detailed description of 1.5 T MRI scanner used for brain imaging in our study and sequence parameters was described elsewhere (Dusek et al. 2020). 3 T MRI scanner was General Electric Signa Architect (GE Healthcare, USA). The MRI protocol included following routine clinical images: T1-weighted (spin-echo [SE], repetition time [TR], 600 ms; echo time [TE], 10 ms), T2-weighted (SE, TR, 6077 ms; TE, 100 ms; voxel resolution, 0.4 × 0.4 × 5 mm^3^), FLAIR (TR, 11.000 ms; TE, 140 ms; inversion time, 2640 ms), T2*-weighted (gradient-echo [GRE], TR, 440 ms; TE, 20 ms; flip angle, 20_), Ax 3D (TR, minimum; TE, 32 ms; flip angle, 15_), Ac T2&Pd FSE (TR, 6077 ms; TE, 100 ms; flip angle, 111_). All images were acquired in the axial plane and covered the entire brain.

Abnormal brain MRI findings typical for WD were defined as: T2/FLAIR hyperintensities in putamen, caudate nucleus, thalamus, mesencephalon, pons; T2/T2*/SWI [Susceptibility weighted imaging] hypointensities in globus pallidus, putamen, caudate nucleus, thalamus, dentate nucleus; atrophy (assessed on T1 weighted sequences) cortical, central, cerebellar (EASL 2012; Dusek et al. 2020). The presence or absence of brain pathology were scored as: (0) no abnormality or (1) present changes in signal intensity or presence of atrophic changes. Brainstem was defined as mesencephalon and pons. We did not assess atrophy in pons and mesencephalon. We also did not use any scale for the evaluation of brain abnormalities.

### Blink reflex

During the BR examination, patients were laying on a comfortable couch in a darkened and quiet room, relaxed. BR was tested according to the standard procedure (Kimura 2001), using the Viking EMG/EP system (Natus, USA).

BR was recorded simultaneously from the right and left orbicularis oculi muscles. Surface stimulation electrodes were placed over the exit of each supraorbital nerve. We used stimuli with duration of 0.2 ms and intensity of 20–30 mA. The single electrical stimulus was repeated 4–6 times with 30 s to 1-min intervals to avoid habituation.

Responses of the orbicularis oculi muscles were recorded by disposable, 1.5 cm diameter surface electrodes placed on both sides of the skin over the orbicularis oculi muscles. Electrodes were placed below the lower lid localized on the outer edge of the orbiculi. The reference electrode was placed on the area of the chin.

We compared results of WD patients with normal values for BR used in our laboratory. Normal R1 latency was considered to be 10.4 ± 2.2 ms and 10.6 ± 2.8 ms for patients aged 21–40 and 41–73 years, respectively. Abnormal R2 latencies were defined as an absent R2 response or values that exceeded normal values for ipsilateral R2 latencies (29.8 ± 4.4 ms and 30.4 ± 5.6 ms for patients aged 21–40 and 41–73 years, respectively) and contralateral R2 latencies (32.5 ± 7.4 ms and 33.4 ± 7.8 ms for patients aged 21–40 and 41–73 years, respectively). For R2 latencies, interside differences more than 5 ms were considered abnormal.

### Statistical analysis

Descriptive statistics were used to present the data as mean ± standard deviation (SD), median and interquartile ranges (IQR), counts and percentages. Continuous variables were compared between patient subgroups with the *t* test or the Mann–Whitney test. Count variables were compared between patient subgroups with the Chi-squared test with continuity correction. Bivariate correlations were analyzed with the Spearman correlation coefficient. *P* < 0.05 was considered statistically significant. All calculations were carried out using JASP software (version 0.12.2).

## Results

### Participants

In total, 44 treatment naïve patients and 66 treated patients were recruited (Table 1). There were slightly more females (54.5%, treatment naïve; 53.0%, treated) than males. Similar proportions of patients in each group had hepatic symptoms (65.9%, treatment naïve; 69.7%, treated), and typical brain MRI abnormalities (59.1%, treatment naïve; 60.6%, treated). Median disease duration was 1 (0.4–20) vs. 10 (5–17) years, in treatment naive and treated patients, respectively. Patients with newly diagnosed untreated WD were younger (32 vs. 37 years, *p* = 0.044), more frequently had abnormal liver enzymes, gamma-glutamyl transferase (GGT) and higher ceruloplasmin (13.4 vs. 7.4, *p* = 0.004) and serum copper levels (63.7 vs. 32.7, *p* = 0.004). Treatment naïve patients more frequently had K–F rings (50.0% vs. 22.4%, *p* = 0.021) and higher punctuation in UWDRS total score (18 vs. 7, *p* = 0.058) and Part II (1.5 vs. 0, *p* = 0.03) compared with treated patients (Table 1).

**Table 1:** General characteristic of the studied group

	Treatment naive (*n* = 44)	On treatment (*n* = 66)	*p* value
Age, mean ± SD	32.25 ± 9.91	36.95 ± 13.01	0.044
Women, *n* (%)	24 (54.5)	35 (53.0%)	0.876
Neurologic symptoms, *n* (%)	27 (61.4)	30 (45.5)	0.479
Hepatic symptoms, *n* (%)	29 (65.9)	46 (69.7)	0.804
Psychiatric symptoms, *n* (%)	3 (6.8)	10 (15.2)	0.185
Any typical MRI changes, *n* (%)	26 (59.1)	40 (60.6)	0.832
Kayser–Fleischer ring, *n* (%)	20 (50)	17 (22.4)	0.021
UWDRS total score in patients with neurological symptoms, median (IQR)	18 (2–32.3)	7 (1.5–14)	0.058
UWDRS Part II, median (IQR)	1.5 (0–14)	0 (0–2)	0.03
UWDRS Part III, median (IQR)	10.5 (2–22.3)	4 (1.5–12)	0.153
Aspartate aminotransferase, median (IQR)	34.5 (25.8–69.4)	21.2 (18.3–28.7)	< 0.001
Alanine aminotransferase, median (IQR)	47.8 (24.6–113.6)	25.4 (19.9–35.7)	< 0.001
Gamma-glutamyltransferase, median (IQR)	75.55 (46.42–133.25)	25.6 (16.0–46.8)	< 0.001
Alkaline phosphatase	96 (83–124)	81 (57–105)	0.004

*IQR* interquartile range, *MRI* magnetic resonance imaging, *SD* standard deviation, *UWDRS* Unified Wilson's Disease Rating Scale

### Blink reflex

There were no statistically significant differences in BR parameters between treatment naïve and treated patients (Table 2). BR abnormalities occurred with similar frequency in both groups (45.5%, treatment naive; 37.9% treated; *p* = 0.429).

**Table 2:** Blink reflex values for treatment naïve and treated patients at baseline

Value	Treatment naïve Median (IQR)	On treatment Median (IQR)	*p* value
dex R1 lat	10.8 (10.2–11.4)	11 (10.38–12)	0.162
dex R1 amp	105 (57.5–129.5)	95 (54.8–147.3)	0.964
dex R2i lat	32.8 (30.25–36.8)	31.4 (29.1–35.7)	0.141
dex R2i amp	239.5 (178.5–296.5)	252 (194.3–333.8)	0.352
dex R2c lat	34.4 (30.9–38.7)	32.4 (30.4–37.1)	0.201
dex R2c amp	219.5 (145.3–310.5)	199.5 (143–280.8)	0.571
sin R1 lat	10.8 (10.4–11.475)	11 (10.5–11.8)	0.146
sin R1 amp	106.5 (55.5–157)	100 (73–159)	0.655
sin R2i lat	32.4 (29.9–36.8)	31.1 (29.5–35.4)	0.173
sin R2i amp	268 (194.25–354.8)	259.5 (203–316.8)	0.604
sin R2c lat	34.2 (31–38)	32.2 (30.2–36.8)	0.202
sin R2c amp	205.5 (155.8–261.5)	213.5 (147.3–293.5)	0.818

*amp* amplitude, *c* contralateral, *dex* right, *i* ipsilateral, *lat* latency, *sin* left

Any abnormal parameters in BR were observed in 45.5% treatment-naïve patients and 37.9% treated patients (*p* = 0.429). Abnormal findings in any of the BR parameters were more frequent in WD patients with neurological than non-neurological presentation (57.5% vs. 28.6%, *p* = 0.002), in those with typical abnormalities in brain MRI (50% vs. 24.4%, *p* = 0.009), or in those with a K–F ring (73.0% vs. 21.5%, *p* < 0.001).

Longer median R1 and R2 latencies, both ipsilateral and contralateral, were significantly more frequently observed in patients with neurological than non-neurological WD, in those with typical brain MRI WD abnormalities and in those with a K–F ring (Table 3).

**Table 3:** Factors affecting R1 and R2 latencies

Value	Neurological presentation Median (IQR)			Typical brain MRI changes Median (IQR)			Kayser–Fleischer ring Median (IQR)		
	Yes	No	*p* value	Yes	No	*p* value	Yes	No	*p* value
dex R1 lat	11.8 (10.7–12.2)	10.7 (10.2–11.4)	0.002	11.2 (10.4–12)	10.7 (10.2–11.8)	0.092	11.4 (10.8–12.5)	10.7 (10.2–11.8)	0.03
dex R1 amp	90 (48–138)	100 (56.5–150.5)	0.757	86.5 (48.5–128)	111 (66–161)	0.121	88 (45–137)	102 (63.75–149.5)	0.272
dex R2i lat	34.6 (28.8–34.0)	31.0 (30.5–37.5)	0.004	33.2 (30–36.7)	31 (28.8–33)	0.007	36.6 (32.2–38.4)	30.8 (28.8–33)	0.00
dex R2i amp	236 (187–324.5)	251 (191.5–327.4)	0.706	243 (192.7–325.7)	245 (191–326)	0.89	229 (173–303)	258 (200–342)	0.048
dex R2c lat	35.8 (31.5–39.1)	32.1 (30.2–35.3)	0.003	34.9 (31.2–38.8)	32.6 (30.2–35.2)	0.006	38.4 (32.2–40.6)	31.6 (30.2–34.4)	0.00
dex R2c amp	200 (140.5–282)	209 (140.5–282)	0.323	186 (131–280)	228 (172–336)	0.039	200 (126–243)	217 (155.5–331.5)	0.066
sin R1 lat	11.4 (10.9–12)	10.7 (10.9–12)	0.00	11.1 (10.7–11.8)	10.6 (10.3–11.2)	0.009	11.2 (10.3–12)	10.8 (10.4–11.4)	0.339
sin R1 amp	90.5 (59.3–151.2)	108 (70–163.5)	0.325	89 (58.5–152.5)	123 (83–169)	0.143	98 (63.5–146)	105 (70–162)	0.359
sin R2i lat	34.2 (30.9–37.5)	30.8 (29.2–33.4)	0.002	33.4 (29.9–36.3)	30 (29.4–32.4)	0.007	36 (31.4–38.2)	30 (29–32.4)	0.00
sin R2i amp	262 (195–330.5)	270 (199–331.5)	0.854	264 (192.2–328.5)	268 (217–350)	0.385	262 (192–308)	268 (200–350)	0.254
sin R2c lat	35.2 (31.8–38.3)	32 (30.2–35.9)	0.011	35.2 (31.6–38)	31.8 (30.2–34.8)	0.01	37.8 (32.4–40.8)	31.7 (30–35)	0.00
sin R2c amp	197 (151–260.5)	212 (149–312)	0.422	200 (148–292)	224 (152–270)	0.636	183 (147–224)	232 (157.5–321.5)	0.009

*amp* amplitude, *c* contralateral, *dex* right, *i* ipsilateral, *lat* latency, *MRI* magnetic resonance imaging, *sin* left

Extended R1 (right R1—*p* = 0.554, left R1—*p* = 0.081) and R2 latencies (right R2 ipsilateral—*p* = 0.317, right R2 contralateral—*p* = 0.364, left R2 ipsilateral—*p* = 0.079, left R2 contralateral—*p* = 0.151) did not correlate with UWDRS results in patients with neurological presentation. Similarly, the scores of disease-related disability (UWDRS Part II) and neurological examination (UWDRS Part III) did not correlate with abnormal R1 and R2 latencies of BR in patients with neurological presentation (data not presented). Median and interquartile range in neurological patients were as follows: total UWDRS score 8.5 (2–21.5), UWDRS Part II score 0 (0–3), UWDRS Part III score 7 (2–19).

Extended R1 (right R1—*p* = 0.044, left R1—*p* = 0.063) and R2 latencies (right R2 ipsilateral—*p* < 0.001, right R2 contralateral—*p* < 0.001, left R2 ipsilateral—*p* < 0.001, left R2 contralateral—*p* = 0.001) correlated with abnormal MRI signal in brainstem in our cohort. Moreover, extended R2 latencies (right R2 ipsilateral—*p* < 0.001, right R2 contralateral—*p* = 0.001, left R2 ipsilateral—*p* < 0.001, left R2 contralateral—*p* = 0.001) but not R1 latencies (right R1—*p* = 0.141, left R1—*p* = 0.172) correlated with abnormal MRI signal in pons.

## Discussion

Our study indicates that an abnormal BR is common in patients with WD. Several reports suggest that BR may be abnormal in patients with some neurodegenerative disorders with various etiology and localization of lesions (Yavuz et al. 2015; Szmidt-Salkowska et al. 2016; de Tommaso et al 2001; Valls-Solé et al. 2004). Patients with dementia with Lewy bodies (DLB) have also been reported to have abnormalities in BR (Anzellotti et al. 2008; Bonanni et al. 2007). In a study including 26 patients with DLB, 26 with MSA, 26 with PD, 20 with Alzheimer disease, and 20 with PSP compared with 30 healthy controls, BR values were significantly delayed only in DLB patients compared with all other groups (*p* < 0.001). In more than half (53.8%) of DLB patients, BR latencies exceeded two SDs of the control mean (Bonanni et al. 2007). These studies suggest that not only the brainstem, basal ganglia, but also cortical dysfunction may affect the interpretation of BR abnormalities.

In the literature, there is only one study published in 1994, which evaluated BR in ten WD patients and their ten family members (Chu 1994). In this study, R1 and R2 latencies as well as R2 duration were prolonged in the patient group, but not in the healthy family group, which confirmed brainstem dysfunction in WD patients. Our results are partly consistent with this study. However, we observed not only delayed R2, but also R1 latencies in our cohort of WD patients. This may suggest that reticular brainstem pathways, including midpons are affected in WD, even in those without clinical symptoms.

Prolonged R1 and R2 latencies in our patients could indicate dysfunction of reticular brainstem pathways or alternatively in the circuit linking the basal ganglia with pons and whole brainstem. Indeed, we found correlation between delayed R1 and R2 latencies and abnormal findings in brainstem and abnormal R2 latencies in pons (which are mediated between spinal tract of trigeminal nerve in the ipsilateral pons and medulla and interneurons forming connections to the ipsilateral and contralateral facial nuclei) in our cohort. We may speculate that copper depositions are also located in those pathways and cause demyelination there. Coincidence of abnormal R1 and R2 latencies and the presence of neurological symptoms, K–F ring and typical changes in MRI may confirm the role of copper deposits in the regulatory circuits controlling BR. Notably effective anti-copper treatment may lead to resolution of K–F ring, brain MRI lesions and frequently reduction or even resolution of neurological symptoms (Czlonkowska et al. 2018). In the treated group, we observed slightly less frequent prolongation of R1 and R2 latencies, when compared to treatment naïve patients. Hence, it can be speculated that BR parameters may also improve with effective anti-copper treatment. However, there are currently no studies supporting this claim.

We did not observe correlation between abnormal BR parameters and UWDRS scores. However, this scale may have some limitations. The questionnaire does not interrogate the exact reasons underlying potential impairments of the activities of daily living. Moreover, the minimal score in this scale may not adequately capture all neurological disability in patients with WD especially if patients have minor abnormalities (Volpert et al. 2017). Low UWRDS total score as well as parts II and III may also partly explain lack of such correlation in our cohort. Moreover, we may not exclude that BR abnormalities may precede the symptoms and be present in subclinical damage to the nerve pathways.

To our best knowledge, this is only the second study to examine BR in WD patients. An additional advantage of our study is that patients previously treated and treatment naïve were separately analyzed and that our cohort consisted of 110 patients, which is a large group for this rare disease.

## Limitations

Our study has some limitations. First, clinicians performing BR examination were not blinded to the results of the clinical evaluation, as well as laboratory, brain MRI findings and the presence of K–F rings. Second, we examined only patients with mild deficit to ensure their cooperation during the examination. Third, we did not compare WD patients results of BR with control group. We may expect that patients with severe neurological or hepatic abnormalities may have more severe reticular brainstem pathways dysfunction. Hence, further studies evaluating BR should focus on patients with severe WD.

## Conclusions and future perspectives

To conclude, abnormal findings in BR seem not to differ significantly between previously treated and treatment naïve patients with WD. If present, abnormal BR parameters were more frequently associated with the presence of neurological symptoms, K–F ring and typical for WD abnormal findings in brain MRI. Further investigations using BR should be performed in a larger group of patients with WD especially those with more severe neurological and hepatic deficits who were not investigated in our study. It would be also reasonable to investigate if effective anti-copper treatment contribute to improvement or resolution of BR abnormalities.
